# Metabolic Changes in Seed Embryos of Hypoxia-Tolerant Rice and Hypoxia-Sensitive Barley at the Onset of Germination

**DOI:** 10.3390/plants10112456

**Published:** 2021-11-14

**Authors:** Jayamini Jayawardhane, M. K. Pabasari S. Wijesinghe, Natalia V. Bykova, Abir U. Igamberdiev

**Affiliations:** 1 Department of Biology, Memorial University of Newfoundland, St. John’s, NL A1C 5S7, Canada; mkpswijesing@mun.ca; 2 Department of Botany, Faculty of Science, University of British Columbia, Vancouver, BC V6T 1Z4, Canada; 3 Morden Research and Development Centre, Agriculture and Agri-Food Canada, Morden, MB R6M 1Y5, Canada; natalia.bykova@agr.gc.ca

**Keywords:** *Oryza sativa*, *Hordeum vulgare*, hypoxia tolerance, nitric oxide, imbibition, reactive oxygen species, ATP/ADP ratio

## Abstract

Rice (*Oryza sativa* L.) and barley (*Hordeum vulgare* L.) are the cereal species differing in tolerance to oxygen deficiency. To understand metabolic differences determining the sensitivity to low oxygen, we germinated rice and barley seeds and studied changes in the levels of reactive oxygen species (ROS) and reactive nitrogen species (RNS), activities of the enzymes involved in their scavenging, and measured cell damage parameters. The results show that alcohol dehydrogenase activity was higher in rice than in barley embryos providing efficient anaerobic fermentation. Nitric oxide (NO) levels were also higher in rice embryos indicating higher NO turnover. Both fermentation and NO turnover can explain higher ATP/ADP ratio values in rice embryos as compared to barley. Rice embryos were characterized by higher activity of *S*-nitrosoglutathione reductase than in barley and a higher level of free thiols in proteins. The activities of antioxidant enzymes (superoxide dismutase, ascorbate peroxidase, monodehydroascorbate reductase, dehydroascorbate reductase) in imbibed embryos were higher in rice than in barley, which corresponded to the reduced levels of ROS, malonic dialdehyde and electrolyte leakage. The observed differences in metabolic changes in embryos of the two cereal species differing in tolerance to hypoxia can partly explain the adaptation of rice to low oxygen environments.

## 1. Introduction

Rice (*Oryza sativa* L.) and barley (*Hordeum vulgare* L.) are economically important cereal crops. Apart from being the staple foods in many countries, rice and barley are also ideal plant models for studying monocot seed germination because of the availability of genomic resources with annotated reference genomes and well-studied physiological, morphological and metabolic traits [1,2]. Rice is an anoxia/hypoxia tolerant species, while barley is hypoxia/anoxia intolerant [3], which makes these species important for elucidating the genetic, physiological and biochemical background of hypoxia tolerance. Since the germinating seeds of all cereals and many other plants are highly hypoxic upon imbibition and before radicle protrusion [4], we used rice and barley as contrasting plant species to study the differences in their metabolism in order to explain the metabolic basis of coping with hypoxia tolerance at the early stages of germination.

Seed germination is a vital stage in the plant life cycle, and it begins with seed rehydration and imbibition [5]. In general, the germination process can be distinguished by three major phases, which include rapid water uptake by a dry seed upon imbibition (phase I), reactivation of metabolism (phase II), and radicle protrusion (phase III) [4]. The second phase is the most critical stage where important physiological and biochemical processes that initiate the germination process reactivate [6]. Due to imbibition, the cell wall enlarges, the seed coat becomes softened [7], and the water availability directs the enzymatic hydrolysis of proteins, lipids and carbohydrates, and the transportation of metabolites [8].

Seed germination and dormancy are under the control of both genetic and biochemical processes [9]. Weakening of the endosperm during germination via by α-xylosidase activity, biosynthesis of xyloglucan in the endosperm, arrangement of cutin coat in the endosperm-testa interface play critical roles in determining dormancy and germination [9]. Some structures of the seed, such as seed coat (or testa), act as a physical barrier for gas exchange [10]. Due to the resumption of respiratory activity following imbibition, the oxygen content in the seed tissue is rapidly diminished (reviewed in [11]). Therefore, the supply of the oxygen through the seed coat to the embryo becomes limited, which generates the hypoxic environment in the seed [12]. Consequently, aerobic respiration in the seed is suppressed and anaerobic respiration is developed to maintain the energy status in cells [13]. Initiation of the fermentation process under hypoxic condition is considered as an adaptive mechanism for ATP synthesis [14,15]. The increased ethanol fermentation in seeds is linked with oxygen deprivation and is catalyzed primarily with the participation of alcohol dehydrogenase (ADH) [14,16]. Consequently, the ATP level and energy charge inside seeds remain high during early germination [17,18].

Moreover, the balance between reactive nitrogen species (RNS) that include nitric oxide (NO) and reactive oxygen species (ROS), acting as the important signaling substances under stress, plays a crucial role in breaking dormancy of seeds and induction of germination [19,20,21,22,23]. Imbibition induces the formation of ROS and RNS [21,24] and leads to the changes in thiol redox-sensitive seed proteome [25,26]. Hypoxic environment within the seed triggers the production of NO under the seed coat [27]. ROS are formed during the restarting of metabolism by the increased oxidative processes leading to the activation of electron transport, in particular at the levels of mitochondrial electron transport chain, of the plasma membrane NADPH oxidase, xanthine oxidase and peroxidases as part of an oxidative burst during rehydration [28]. Antioxidant systems and proteins that scavenge reactive radicals and NO reduce oxidative stress damage in seeds to prevent loss of germination capacity [29,30] and these biochemical events are activated immediately upon rehydration [31,32,33,34,35].

In this study, we evaluated metabolic differences that control the sensitivity to oxygen deficiency in germinating rice and barley seeds. Changes in the levels of ROS and RNS, in activities of the enzymes involved in their generation and scavenging were determined, and parameters measuring cell damage such as malonic dialdehyde and electrolyte leakage were assessed. The observed differences in metabolic responses in embryos of the cultivars of two cereal species differing in tolerance to hypoxia are discussed in relation to the development of mechanisms of metabolic adjustments to low oxygen conditions occurring in germinating seeds.

## 2. Results

### 2.1. Germination Rates of Rice and Barley Seeds

**Figure 1:**
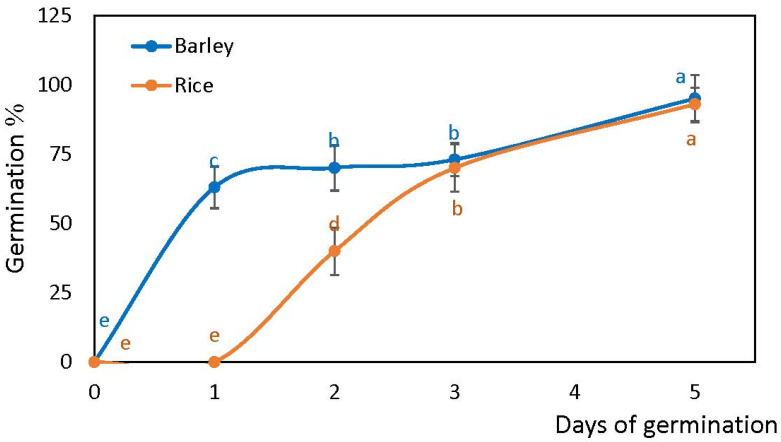
Germination of rice and barley seeds. Germination rates of two species within five days of imbibition. Vertical bars represent standard deviations (*n* = 3). Different letters indicate significant differences between the two species and the time points at *p* < 0.05, (one-way ANOVA test, Tukey comparison).

Barley seeds started radicle protrusion at 15 h while in rice the protrusion of radicle was delayed until 48 h of post-imbibition. Both species showed a similar germination percentage at the end of five days after imbibition (barley 97%, rice 93%). However, the rate of germination in rice at the initial stage was lower than in barley. After three days, a similar germination percentage was reached in both species, and was comparable until the end of five days of germination assay (Figure 1).

### 2.2. Alcohol Dehydrogenase Activity and the Level of Adenylates during Germination

**Figure 2:**
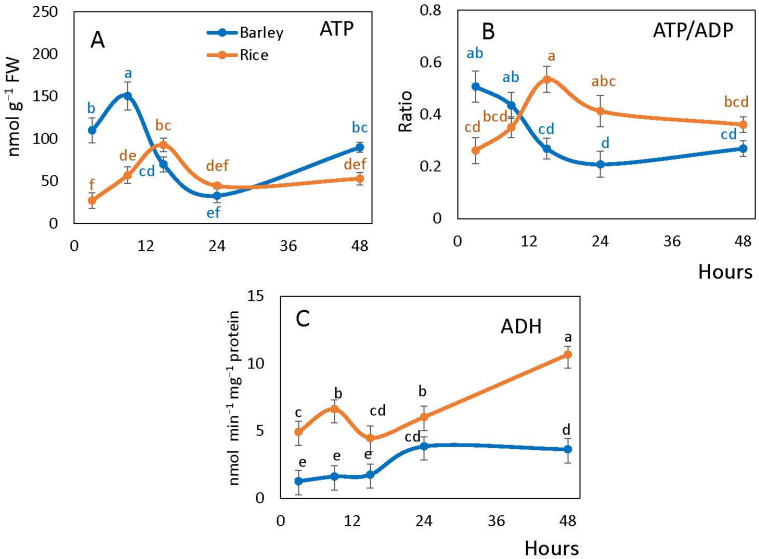
Total ATP (**A**), ATP/ADP ratio (**B**) and ADH activity (**C**) in barley and rice seeds following imbibition. Vertical bars represent standard deviations (*n* = 3). Different letters indicate significant differences between the two species and the time points at *p* < 0.05, (one-way ANOVA test, Tukey comparison).

Embryonic ATP content exhibited the initial fluctuation in both species (Figure 2A). In barley, the peak of ATP level was observed at 9 h from the start of imbibition while in rice at 15 h. The following decline was sharp in barley and smoother in rice. Further increase of ATP content by 48 h was pronounced in barley but was not observed in rice (Figure 2A).

The ATP/ADP ratio generally followed a similar pattern to the ATP content in rice, but in barley seed embryos it gradually declined upon imbibition and then stabilized. In rice embryos, the ATP/ADP ratio increased from the values of ~0.2 lower than in barley at 3 h peaking at ~0.25 higher than in barley at 15 h, and then slightly decreased (Figure 2B).

ADH activity in the embryos was significantly higher in rice than barley. In rice, it markedly increased at 9 h after imbibition followed by a drop at 15 h, after which it continued to increase significantly. On the contrary, ADH activity in barley remained constant for 15 h and then increased by 24 h (Figure 2C).

### 2.3. Nitric Oxide, Free Thiols, S-Nitrosoglutathione Reductase and S-Nitrosylation

**Figure 3:**
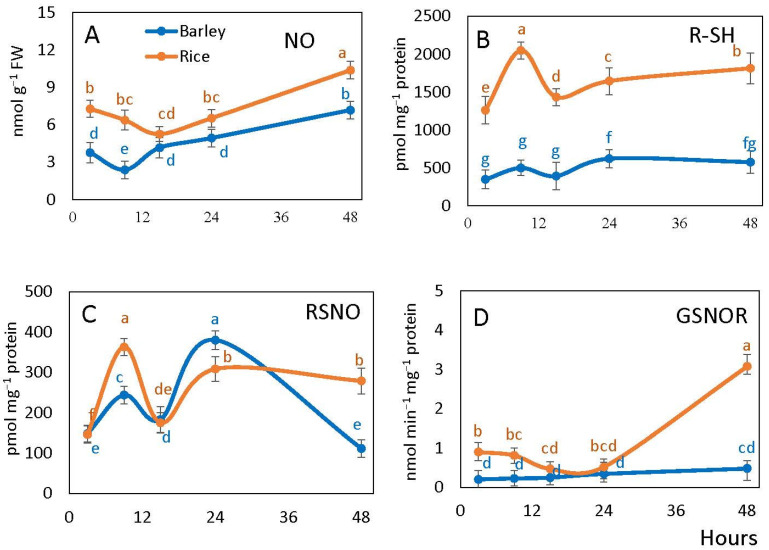
Levels of NO (**A**), free thiols (R-SH) (**B**), *S*-nitrosylation (RSNO) (**C**) and *S*-nitrosoglutathione (GSNOR) activity (**D**) in barley and rice embryos following imbibition. Vertical bars represent standard deviations (*n* = 3). Different letters indicate significant differences between the two species and the time points at *p* < 0.05, (one-way ANOVA test, Tukey comparison).

NO content in embryos during germination remained higher in rice than in barley throughout the whole time of observation, the difference was not significant only at 15 h. It decreased until 15 h of imbibition in rice and until 9 h in barley, and then continuously increased (Figure 3A).

Free thiol (RSH) content of embryos was essentially higher in rice embryos than in barley embryos. In rice, the content of thiols exhibited an increase at 9 h after imbibition followed by a decrease at 15 h and further increase to 24 h. In barley, only a slight increase was observed from 15 to 24 h. Between 24 to 48 h of imbibition, the measured R-SH content remained stable in barley and slightly increased in rice (Figure 3B).

The content of *S*-nitrosylated (RSNO) groups exhibited significant fluctuations over time points in both species. It increased sharply in rice and more moderately in barley at 9 h, and then decreased by 15 h of imbibition. A further increase was observed at one day of imbibition, after which RSNO level remained stable in rice and decreased in barley (Figure 3C).

*S*-nitrosoglutathione reductase (GSNOR) activity of seed embryo was higher in rice than in barley in the first 9 h and by 48 h after imbibition. The activity level was stable in barley during the entire period of observation, while in rice it decreased by 15 h and then increased sharply from 24 to 48 h (Figure 3D).

### 2.4. Reactive Oxygen Species, Lipid Peroxidation and Electrolyte Leakage in Rice and Barley Seeds

**Figure 4:**
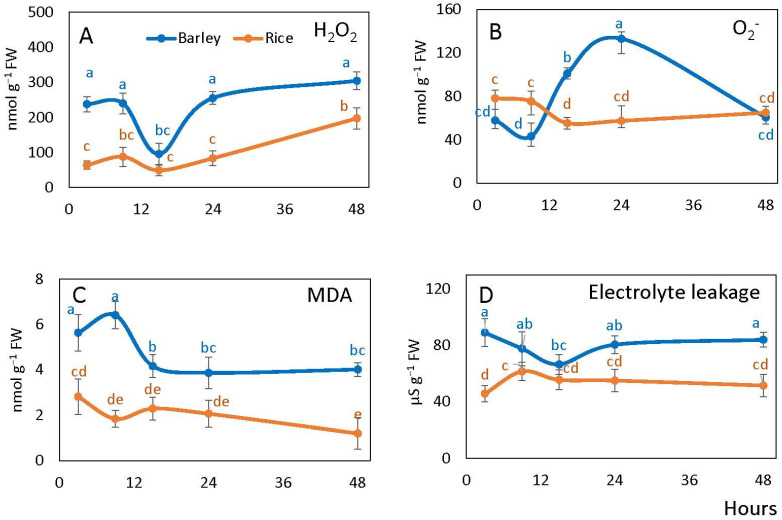
Barley and rice seed embryonic hydrogen peroxide (H_2_O_2_) (**A**), superoxide anion (**B**), lipid peroxidation in terms of MDA (**C**) and seed electrolyte leakage (**D**) following imbibition. Vertical bars represent standard deviations (*n* = 3). Different letters indicate significant differences between the two species and the time points at *p* < 0.05, (one-way ANOVA test, Tukey comparison).

The level of hydrogen peroxide (H_2_O_2_) in embryo was significantly higher in barley than in rice during all periods of observation, except the 15 h point. In barley, apart from the steep decrease from 9 to 15 h corresponding to the time before radicle protrusion, the level remained constant and exceeded H_2_O_2_ level in rice by 2–4 times. Rice embryo showed a generally stable H_2_O_2_ level until 24 h, followed by the increase to 48 h (Figure 4A).

Embryonic superoxide anion content in rice was slightly higher than in barley at 9 h after imbibition, then decreased by 15 h and did not change significantly in the subsequent hours. In barley, superoxide levels increased by 15 h peaking at 24 h at the levels threefold higher than at the beginning of imbibition, followed by further decline to 48 h (Figure 4B).

Malondialdehyde (MDA) levels in the embryos remained significantly (2–3 times) higher in barley than in rice after imbibition being the highest at 3 and 9 h of imbibition (before radicle protrusion). MDA levels in rice did not exhibit significant changes over the whole period (Figure 4C).

Electrolyte leakage of germinating barley seeds was also significantly higher in barley than in rice during almost all period of observation (except the 15 h point). The levels slightly declined in barley until 15 h and then slightly increased to 48 h. In rice seeds, the changes in the level of electrolyte leakage were statistically insignificant over the germination period tested except the initial slight increase (Figure 4D).

### 2.5. Antioxidant Enzyme Activities in Rice and Barley Seeds

**Figure 5:**
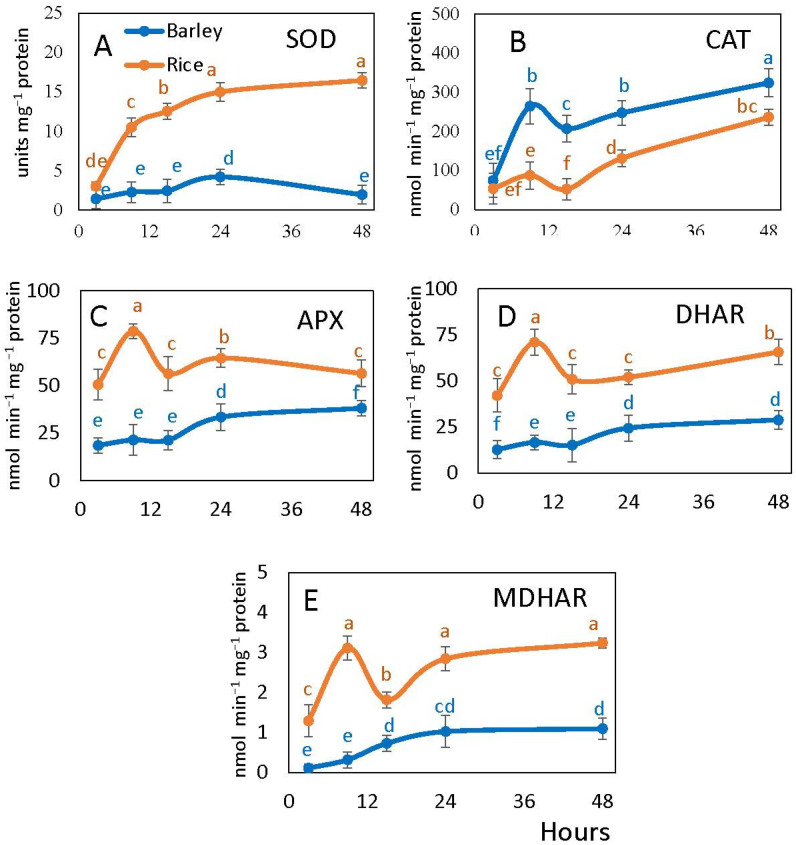
Embryonic antioxidant enzyme activities of barley and rice seeds following imbibition: superoxide dismutase (SOD) (**A**), catalase (CAT) (**B**), ascorbate peroxidase (APX) (**C**), dehydroascorbate reductase (DHAR) (**D**), monodehydroascorbate reductase (MDHAR) (**E**). Vertical bars represent standard deviations (*n* = 3). Different letters indicate significant differences between the two species and the time points at *p* < 0.05, (one-way ANOVA test, Tukey comparison).

All studied antioxidant enzymes except catalase exhibited higher activity in rice than in barley (Figure 5). SOD activity increased sharply in rice embryo during germination reaching 5–10 times higher values than in barley where it changed only slightly with the two-fold increase by 24 h (after radicle protrusion) (Figure 5A). Catalase activity strongly increased in barley embryos at the onset of germination, while in rice embryos it was always lower and increased only at 24 h of imbibition (Figure 5B).

APX, MDHAR and DHAR activities were consistently higher in rice than in barley exhibiting an increase at 9 h and decrease at 15 h. In barley embryos, they were lower with a smooth increase of APX and DHAR by 24 h (upon radicle protrusion) and the earlier continuous increase of MDHAR (Figure 5C–E).

## 3. Discussion

### 3.1. Seed Germination Phases and Hypoxic Conditions under Seed Coat

Seed imbibition triggers many biochemical and cellular processes associated with germination [6,36]. As water is taken up by the dry seed, it triggers the transition of seed to germination which completes with the emergence of embryonic radicle tissues from the seed coat [8]. Germination of cereals is controlled by complex signalling networks including both internal and external cues [23]. Phytohormones such as ABA and GA act as the hubs that connect internal and external signals, controlling germination antagonistically, whereas other phytohormones, carbohydrates, ROS, NO, microRNAs, light, and temperature also affect germination at the transcriptional, translational, and post-translational levels [23]. Bewley [4] observed radicle protrusion in rice at 50 h of imbibition and Ma et al. [35] observed radicle protrusion in barley at 12–15 h of imbibition. Our results demonstrate that barley seeds started radicle protrusion at 15 h of imbibition, while in rice it only happened between 24 and 48 h after onset of imbibition (Figure 1).

According to Gruwel et al. [37], barley seeds hydrate rapidly during very early imbibition and the absorbed water is mainly confined to the embryo tissue. A triphasic model was introduced to reflect the increase of total water content during germination [4]. During the first 20 h of imbibition (Phase I), rice seeds increased in weight rapidly without significant morphological changes [4]. Phase II showed a stable plateau until 50 h where the coleoptiles elongated [4]. Another rapid water uptake stage and radicle protrusion were shown to take place in Phase III [38].

While Phase I is characterized by the dramatic increase in respiration rates in seeds, the internal oxygen concentration due to the low permeability of seed coat is rapidly depleted [4,11]. The seed becomes hypoxic at Phase II for a prolonged period of time in rice, while in barley this phase is significantly shorter. A longer duration of the hypoxic phase in rice means the persistence of efficient anaerobic metabolism in this plant, while in barley, lower resistance to oxygen deficiency requires a fast completion of this phase and accelerated transition to Phase III initiated by radicle protrusion. From this point of view, the comparison of metabolism in rice and barley during seed germination can reveal essential metabolic differences at this stage related to hypoxia sensitivity in plants. Below we discuss these differences in relation to fermentation, nitric oxide metabolism, production and scavenging of ROS and RNS in the overall context of energy metabolism of the germinating seeds.

### 3.2. Respiration and Energy Availability in the Embryos of Imbibed Seeds

Due to a rapid depletion of oxygen in seed tissues upon imbibition, oxidative respiration becomes limited, resulting in the depleted energy status of early germinating seeds [39,40,41]. According to He et al. [42], aerobic respiration in rice seeds is quite low during the first 48 h of imbibition due to the lack of functional mitochondria. However, seed ATP levels and energy charge remain elevated because of the glycolytic fermentation and/or other adaptive and alternative mechanisms for energy generation [17,18,43]. Anaerobic respiration pathways, such as fermentation, might be the main source of energy at the early stage of germination of rice seeds [38,44]. Fermentation in terms of ADH activity was higher in rice than in barley and it increased continuously in the first nine hours and then from 15 h towards 48 h when all rice seeds become germinated (Figure 2C). This indicates more efficient anaerobic metabolism in rice than in barley and explains the observed fact that the germination of rice seeds can take more time, during which oxygen becomes almost fully depleted. During this time (starting from 15 h), rice seeds can maintain higher ATP/ADP ratio than barley seeds despite of the delayed radicle protrusion. At 24 h of imbibition, when most barley seeds are germinated and rice seeds are not, the ATP/ADP ratio remains higher in rice. In addition to the glycolytic fermentation, alternative pathways such as the phytoglobin-NO cycle, can contribute to the maintenance of high energy state under the conditions of hypoxia [45]. Further, phytoglobin (*Pgb1*) gene expression is essential to maintain redox and energy balance before radicle protrusion, when seeds experience low internal oxygen concentration [46].

### 3.3. Nitric Oxide and ROS Scavengers in Embryo of Imbibed Seeds

Seeds produce ROS such as H_2_O_2_, O_2_^−^, and hydroxyl radicals, and RNS, such as NO, during imbibition [21,24,47,48,49]. ATP production under the conditions of oxygen deficiency can be associated not only with the glycolytic fermentation but also with the phytoglobin-NO cycle [50]. NO is scavenged by class 1 phytoglobin that is expressed within 2 h after imbibition of seeds [17,45,51,52,53]. Our results revealed that the NO level was higher in rice than in barley embryo (Figure 3A), suggesting that the phytoglobin-NO cycle could be more active in rice, and a higher ATP/ADP ratio in rice than in barley at 15–24 h might be related to the activity of this cycle. For rice, it has been shown that the phytoglobin-NO cycle and the mitochondrial alternative oxidase play a vital role in anaerobic germination and growth of deep-water rice [54]. A sharper decrease of RSNO (which mostly refers to *S*-nitrosoglutathione) in rice than in barley (Figure 3C) after 9 h may be associated with the direction of NO towards nitrate formation in the phytoglobin-NO cycle instead of *S*-nitrosylation of glutathione and proteins. *S*-nitrosoglutathione is scavenged by GSNOR, which is the class III alcohol dehydrogenase having also the activity of formaldehyde dehydrogenase [55]. From the obtained values of RSNO levels, it can be concluded that a part of the produced NO goes to *S*-nitrosylation, and this process decreases in barley after radicle protrusion, while in rice seeds that protrude radicles later, *S*-nitrosylation remains stable at 24–48 h due to the increased GSNOR activity (Figure 3D). More efficient NO scavenging in rice may explain the observation that free SH groups are present at a higher level than in barley (Figure 3B).

### 3.4. Reactive Oxygen Species and Antioxidant System in Seed Embryo during Germination

Imbibition processes that induce ROS formation (mostly H_2_O_2_) facilitate dormancy decay and promote germination [56,57]. The balance between ROS-producing and ROS-scavenging systems plays a key role in seed germination and dormancy alleviation [21]. The ability of seeds to germinate is linked with the accumulation of a critical level of H_2_O_2_ [26]. Our results show higher H_2_O_2_ levels in barley seed embryo than in rice (equaling only at 15 h) and higher superoxide levels at 15–24 h, which correlates to the earlier onset of germination in barley seeds (Figure 4A,B). A lower NO level in barley embryo may be associated with the involvement of ROS in NO scavenging [22]. Higher ROS levels in barley embryos can also explain the increased values of cell damages in terms of lipid peroxidation and electrolyte leakages (Figure 4C,D). ROS are scavenged by the efficient antioxidant systems [29,30,33], which are activated immediately upon rehydration [31,32]. Lower ROS levels in rice embryos can be explained by higher activities of most antioxidant enzymes (except catalase) (Figure 5). Our results demonstrate that in rice embryos, APX, having higher affinity to H_2_O_2_ [58], is more involved in its scavenging than catalase as compared to barley. While in our study generally lower ROS levels correspond to a slower germination in rice than in barley, this correlation is comparable to the postulation of “oxidative window” for dry seeds resulting in seed dormancy decay and aging [20,59]. Germinating rice seeds are characterized by a lower level of cell damage, higher fermentation and NO turnover rates and higher ATP/ADP ratios. This results in the germination process in rice being less susceptible to stress factors as compared to barley, including the ability to germinate anaerobically.

## 4. Materials and Methods

### 4.1. Seed Germination and Isolation of Embryos

Barley (*Hordeum vulgare* L. cv. Harrington) and rice (*Oryza sativa* L. ssp. *indica,* cv. FR13A) seeds were surface sterilized with 10% NaOCl and washed three times with autoclaved distilled water. Seeds were soaked in sterile deionized water on filter papers in Petri dishes at 25 °C in darkness. The germination rate (total seeds germinated at end of trial/number of initial seeds ×100%) was calculated according to Al-Mudaris [60].

We isolated fresh embryos of imbibed seeds at different hours following imbibition (3, 9, 15, 24, 48 h), froze them in liquid nitrogen and stored at −80 °C for further analysis to investigate the biochemical changes during germination within an extensive time course, from dry seeds to radicle protrusion.

All chemicals, unless indicated otherwise, were obtained from Sigma–Aldrich, St. Louis, MO, USA.

### 4.2. Alcohol Dehydrogenase and Adenylate Ratios

Alcohol dehydrogenase (ADH; EC 1.1.1.1) was measured according to Blandino et al. [61] in the direction of ethanol to acetaldehyde in 50 mM Tris-HCl buffer, pH 8.0, 150 mM ethanol and 2 mM NAD^+^ at 340 nm (ε = 6.22 mM^−1^ cm^−1^).

ATP and ADP were extracted according to Joshi et al. [62] and Yuroff et al. [63] with minor modifications. The tissue powder was lysed on ice in 2.4 M perchloric acid for 60 min and centrifuged for 5 min at 20,000× *g* at 4 °C. The supernatant was neutralized with 4 M KOH, and the ratio ATP/ADP in the neutralized solution was determined according to the manufacturer’s instructions using the EnzyLight^TM^ ADP/ATP Ratio Bioluminescent Assay Kit (ELDT-100) (BioAssay Systems, Hayward, CA, USA). The content of ATP and ADP was determined using ATP and ADP standards.

### 4.3. Nitric Oxide, Free Thiols, S-Nitrosylation and S-Nitrosoglutathione Reductase

NO levels in seed embryos were measured by the hemoglobin (Hb) method at 415 nm (ε = 131 mM^−1^ cm^−1^), as described by Murphy and Noack [64] and Ma et al. [35]. R-SH and RSNO were measured spectrophotometrically by reducing RSNO to R-SH in the presence of ascorbate and then assaying free thiol groups using 5,5′-dithio-*bis* (2-nitrobenzoic acid) (DTNB) at 412 nm [35,65]. *S*-nitrosoglutatione reductase (GSNOR) activity was measured at 340 nm according to Sakamoto et al. [66]. Total soluble protein content was determined according to Bradford [67] using Bradford reagent (Sigma–Aldrich, St. Louis, MO, USA) and Bovine Serum Albumin (BSA) standard at 595 nm.

### 4.4. Hydrogen Peroxide, Electrolyte Leakage and Lipid Peroxidation

H_2_O_2_ content was estimated according to Velikova et al. [68] with modifications. Fresh plant biomass (600 mg) was homogenized in 3 mL 0.1% (*w/v*) TCA and centrifuged at 12,000× *g* for 20 min at 4 °C. Supernatant (300 μL) was mixed with 500 μL of 2 M KI and 200 μL of 10 mM potassium phosphate buffer (pH 7.0). The reaction mixture was incubated for 1 h in the dark at room temperature and the absorbance was recorded at 390 nm. A standard curve was prepared to quantify the H_2_O_2_ content.

Electrolyte leakage from seeds at different hours of post-imbibition was measured according to Tammela et al. [69] with few modifications as described by Agarie et al. [70]. The seed coat was separated and 0.2 g seeds at different hours of post-imbibition was placed in 10 mL of deionized water. The electrical conductivity of the liquid phase was measured by using a handheld portable EC and TDS meter (E-1 TDS and EC meter, Aquasana Inc., Austin, TX, USA) after keeping the tubes with seeds for 24 h in dark conditions [70].

Lipid peroxidation was measured by tracing malondialdehyde (MDA, ε = 156 mM^−1^ cm^−1^) content using thiobarbituric acid (TBA) method as described by Heath and Parker [71].

### 4.5. Superoxide Dismutase, Catalase and Ascorbate-Glutathione Cycle Enzymes

Superoxide dismutase (SOD; EC 1.15.1.1) activity was assayed according to Beauchamp and Fridovich [72] by the inhibition of the photochemical reduction of nitroblue tetrazolium (NBT) at 560 nm. The amount of enzyme causing 50% inhibition of NBT reduction was used to calculate SOD activity. Catalase (EC 1.11.1.6) activity was measured according to the Guilbault [73] using the molar extinction coefficient (ε) for H_2_O_2_ of 43.1 M^−1^ cm^−1^.

The enzymes of ascorbate-glutathione cycle were extracted and assayed as described by Ma et al. [35]. Ascorbate peroxidase (APX; EC 1.11.1.11) was estimated in 50 mM potassium phosphate buffer (pH 7.0) containing 0.5 mM sodium ascorbate and sample extract. The reaction was started by adding H_2_O_2_ (final concentration 1 mM) at 290 nm (ε = 2.8 mM^−1^ cm^−1^). Monodehydroascorbate reductase (MDHAR; EC 1.6.5.4) activity was measured in 50 mM HEPES-KOH buffer (pH 7.6) containing 2.5 mM ascorbate, 0.25 mM NADH, and the extract. The assay was initiated by adding 0.4 U mL−1 of ascorbate oxidase and the reaction was monitored at 340 nm for 3 min (ε = 6.22 mM^−1^ cm^−1^). Dehydroascorbate reductase (DHAR; EC 1.8.5.1) activity was measured in 50 mM HEPES-KOH buffer (pH 7.0) containing 0.1 mM EDTA, 2.5 mM GSH, and the extract. The reaction was initiated by adding freshly prepared dehydroascorbate (final concentration 0.8 mM) (ε = 14 mM^−1^ cm^−1^).

### 4.6. Statistical Analysis

All data were subjected to one-way ANOVA and Tukey’s multiple comparison test by using the Minitab statistical software package (Penn State University Park, State College, PA, USA, 2017). The data in the figures represent the means of three biological repeats and three technical replicates ±SD. Different letters indicate significant differences between the two species and the time points at *p* < 0.05.

## 5. Conclusions

Rice and barley belong to the same plant family but significantly differ in their metabolic processes activity, driving the subsequent germination steps. The embryos of rice seeds possess higher alcohol dehydrogenase activity, indicating more efficient anaerobic fermentation and elevated NO levels corresponding to higher NO turnover rates via the phytoglobin-NO cycle. Both fermentation and NO turnover result in a higher ATP/ADP ratio value in rice embryos prior to radicle protrusion, as compared to barley. The observed changes in seed metabolism following imbibition are due, in particular, to the differences in tolerance to the oxygen deficiency occurring under the seed coat. Radicle protrusion governs the subsequent transition to aerobic metabolism in the embryonic tissue. The balance and the crosstalk between NO, ROS and their scavengers determine the whole germination process under the oxygen depleted conditions of the imbibed seeds and upon aeration after radicle protrusion at the stage of seedling development.

## Data Availability

The datasets generated for this study are available upon request from the corresponding author.
